# Supplementary material for: Easyreporting simplifies the implementation of Reproducible Research layers in R software
Source: PLoS One. 2021 May 10;16(5):e0244122. doi: 10.1371/journal.pone.0244122 (PMC8109797; doi:10.1371/journal.pone.0244122)
Supplement: S1 File — (PDF) [file pone.0244122.s001.pdf]

Supplementary Materials for easyreporting

A Bioconductor package for implementing reproducible research

Dario Righelli* Claudia Angelini†

2021-03-15

1 Abstract

easyreporting is an R/Bioconductor package developed to facilitate the implementation of Reproducible Research (RR) inside other packages/software with requiring low/no knowledge of the R Markdown language.

More in detail, *easyreporting* is an S4 class that schematically represents the structure of a R Markdown file. Therefore, with *easyreporting* an analysis report can be seen as a particular instance (i.e., R object) of the easyreporting class. A series of attributes and methods implemented with the class allow the user to structure the components of the report in the desired way, by adding titles, document sections, comments, code chunks, and so on (see Table S1 for the list of attributes and methods). The R object is step-by-step updated each time a new code chunk is added. At the end of the analysis, the R object can be compiled to produce the typical HTML report of the analysis that can be attached to a publication as supplementary material.

easyreporting can be useful in any data analysis project, but it turns out to be particularly useful in the bioinformatics field, where the complexity of the analyses makes it extremely difficult to trace all the steps and parameters used in the data analysis. Moreover, *easyreporting* can be also used by developers to automatically trace the analysis steps within Graphical User Interfaces (GUIs).

2 General Description

You can also find additional files of this work at the following link such as:

https://github.com/drighelli/easyreporting_supplementary

- this document
  - Rmd file
  - Pdf file
  - Tex file (produced by rmakrdown)
- the files needed to produce the report described in our work
  - R file with the RNA-seq analysis made using easyreporting
  - Rmd report file produced by easyreporting
  - Pdf file of the Rmd compiled report

2.1 Package Installation

easyreporting is available on Bioconductor since version 3.11 (R>=4.0).

To install it, please execute the following code:

*Istituto per le Applicazioni del Calcolo "M. Picone" - Consiglio Nazionale delle Ricerche

†Istituto per le Applicazioni del Calcolo "M. Picone" - Consiglio Nazionale delle Ricerche

Functions	Description	Package Location
importData	wrapper for reading xlsx data file	script/importFunctions.R
applyEdgeREx	performs edgeR Differential Expression test	script/geneFunctions.R
MAedgeRMAPlotEx	shows an MA-plot starting from edgeR results	script/plotFunctions.R
VolcanoPlot	shows a Volcano-plot starting from edgeR results	script/plotFunctions.R
traceAndPlotMAPlot	wrapper for tracing and showing the MAedgeRMAPlotEx	script/plotFunctions.R
traceAndPlotVolcano	wrapper for tracing and showing the Volcano plot into the Shiny GUI	shiny_examples/volcano_
server	Shiny GUI server for the Volcano plot examaple	shiny_examples/volcano_
ui	Shiny GUI ui for the Volcano plot examaple	shiny_examples/volcano_

Table S1: Accessory functions released with the *easyreporting* package.

```
if (!requireNamespace("BiocManager", quietly = TRUE))
    install.packages("BiocManager")

BiocManager::install("easyreporting")
```

After the package is installed, the user needs to load the *easyreporting* package into the R workspace by using the following command

```
library("easyreporting")
```

2.2 Easyreporting Accessory Functions

Supplementary Table S1 lists the additional accessory functions that can be easily imported with the `system.file` as examples of user-defined functions. Note, these functions do not belong to the *easyreporting* package, but they are released within the package as external scripts to show the user how to trace external functions.

3 Easyreporting Bulk RNA-seq Analysis

The following sub-sections are organized to offer an example of how *easyreporting* can be used to create an automatic R Markdown file describing a given bulk RNA-seq analysis. For the sake of generality, we divided the analysis in several code chunks (CC) and we used in the CCs both functions from well-known R packages and user-defined functions.

To perform a complete walkthrough usage example, for each CC, we first show the *easyreporting* CC (section named "code side") with the R code that is needed to be tracked. Then, the same CC is presented as a piece of an R Markdown code (section named "rmarkdown side"). Such a rmarkdown code is auto produced by the *easyreporting* code side CC. Then, finally, the third section of the CC shows how the above R Markdown code appears as a piece of the final report (section named "report side").

The shown code has to be meant as illustrative for better understanding how *easyreporting* can be used to trace code and functions into a bigger project, such as a GUI for data analysis.

3.1 Dataset Description and RNA-seq pipeline

As an illustrative purpose, we consider an RNA-seq dataset (GEO Accession: *GSE60231*) selected from our previous work on CD8+ dendritic T-cells aimed to investigate the differences in the immune response of two different antibodies compared with control.

The dataset contains the raw counts of 37991 genes and is composed of two replicates for each of the three conditions:

- DEC (fd-scaDEC-205 antibody samples)

- E2 (E2 antibody samples),
- UNTR (control samples)

See Costa et al. (2017) for more details.

A typical RNA-seq analysis starts from the mapping of the FASTQ sequences to a reference genome, followed by the gene expression quantification of each sample. The quantification step leads to the so-called raw count matrix, with genes represented on the rows and samples on the columns, i.e., where each matrix element (i,j) contains the number of reads of the gene (i) in the sample (j). Such a phase is the most computationally demanding part and skipped in this example.

For illustrative purposes, here we start the analysis from the raw counts.
The raw count file is released as supplementary data with the *easyreporting* package (file *BMDC_counts_FeaatureCounts.xlsx*). The illustrated pipeline will first load the data, perform some diagnostic plots, filter and normalize the raw count, and visualize the principal component projection. Then, it will perform differential gene expression analysis and depict the results in terms of a Venn diagram and MA-plots. Each phase is described by a specific CC.

The first step requires to initialize the report, then it needs to be updated each time with a new CC, adding sections and comments when necessary/possible. At the end of the analysis, the analyst can compile the final report by using the properly designed method. Each of the above-mentioned phases the *easyreporting* methods will be used.

3.2 Report initialization

After loading the *easyreporting* package in the R environment, the it's necessary to initialize an analysis report by providing the file name (i.e., "rnaseq_report") and the title of the report (i.e., "RNA-seq Analysis Report"). It is also possible to specify an author (i.e., Dario Righelli).

For simplicity, we set-up a project directory path starting from the working directory for our report, but other locations can be chosen by setting the `file.path` parameter.

The initialization is carried out using the function `easyreporting()`. Note that the `filenamePath` and `title` are mandatory parameters, while the `author` is optional.

3.2.1 CC1 code side

To initialize the report, it can be used the following code.

We provide the possibility to add more than one author with some additional information associated.

Moreover, it is possible to specify a latex `bib` file with cited references with the dedicated argument. This file will be processed during the report compilation process.

```
library("easyreporting")
proj.path <- file.path(getwd(), "rnaseq_report")
bioEr <- easyreporting(filenamePath=proj.path, title="RNA-seq Analysis Report",
                       author=c(
                           person(given="Dario", family="Righelli",
                                  email="fake_email@gmail.com",
                                  comment=c(ORCID="ORCIDNUMBER",
                                            url="www.fakepersonalurl.com",
                                            affiliation="Institute of Applied Mathematics, CNR, Naples,
                                            affiliation_url="www.fakeurl.com")),
                           person(given="Claudia", family="Angelini",
                                  comment=c(ORCID="ORCIDNUMBER",
                                            url="www.fakepersonalurl.com",
                                            affiliation="Institute of Applied Mathematics, CNR, Naples,
```

```
                                        affiliation_url="www.fakeurl.com"))
                    ), bibfile="ref.bib")
```

3.2.2 CC1 rmarkdown side

The above CC1 code initializes the object *bioEr* as an instance of the *easyreporting* class and produces the following lines of code that define the header and the options CC inside the “rnaseq_report.rmd” file.

```
% ---
%    title: "RNA-seq Analysis Report"
%    author:
%      - name: " Dario Righelli fake_email@gmail.com "
%        orcid_id: ORCIDNUMBER
%        affiliation: Institute of Applied Mathematics, CNR, Naples, IT
%        affiliation_url: www.fakeurl.com
%        url: www.fakepersonalurl.com
%
%      - name: " Claudia Angelini"
%        orcid_id: ORCIDNUMBER
%        affiliation: Institute of Applied Mathematics, CNR, Naples, IT
%        affiliation_url: www.fakeurl.com
%        url: www.fakepersonalurl.com
%
%    date: "2021-03-15"
%    bibfile: ref.bib
%    output: distill::distill_article
%---
```

3.2.3 CC1 report side

Above CC1 R Markdown side adds into the report the Title, the date time and the author, and gives instructions for producing the HTML file format, as shown in Figure S1.

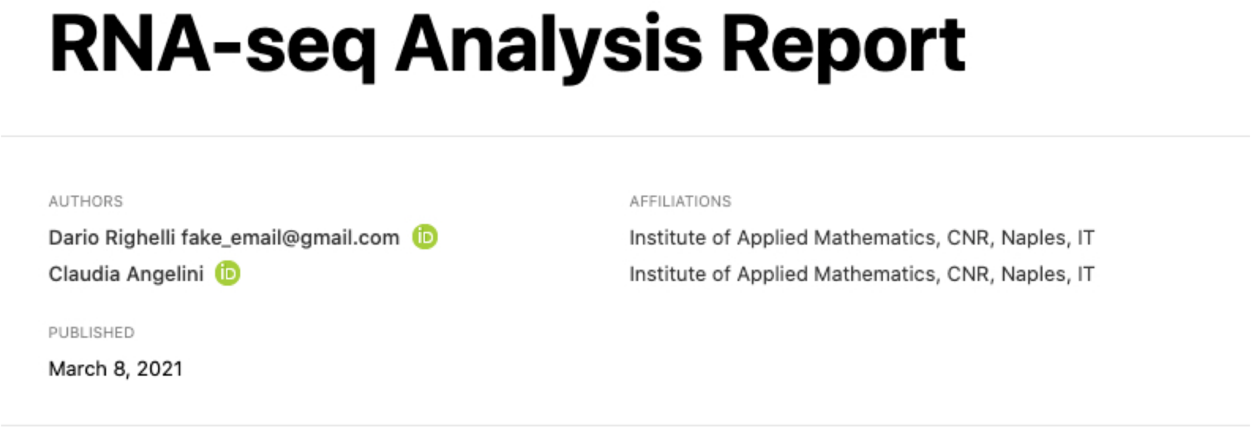

Figure S1: The header produced by the CC1 R Markdown side code.

3.3 Loading Data

Once the report is initialized, it can be added a CC for each step of the analysis. As mentioned in Section General Description, *easyreporting* provides two class methods for adding CCs within a report.

In the first case, it is needed to use the `mkdCodeChunkSt()` to open a new CC. Then, to add the code to the markdown, it can be used the `mkdVariableAssignment()` and/or the `mkdGeneralMsg()` functions, for tracking variables and functions. Finally, the CC can be closed by using the `mkdCodeChunkEnd()` function.

In the second approach the`mkdCodeChunkComplete()` function allows tracing the steps through the `message` parameter.

In both cases, it is possible to organize the report in sections, using the `mkdTitle()` function. Such operations will be repeated for each step of the analysis.

Here, we assume that to read the raw counts a user-defined function named `importData` is already stored in the `importFunctions.R` file available into the package "script" folder (see Supplementary Table S1) and we show as illustrative examples both approaches.

3.3.1 CC2 code side

For the sake of illustration, here we use the first approach for adding the CC. For this purpose, the analyst can use the following code.

```
mkdTitle(bioEr, title="Loading Counts Data")
mkdCodeChunkSt(bioEr, sourceFilesList=system.file("script/importFunctions.R",
                                   package="easyreporting"), isComplete=TRUE)
mkdVariableAssignment(bioEr, "geneCounts", paste("as.matrix(importData(system.file('",
                             "extdata/BMDC_counts_FeatureCounts.xlsx', ",
                             "package='easyreporting')))", sep="\n"), show=FALSE)
mkdGeneralMsg(bioEr, "head(geneCounts, 20)")
mkdCodeChunkEnd(bioEr)
```

For comparative purposes, the above code can be replaced by the following code that implements the second approach. Note that a `c` list of `quote` expressions can be used to specify multiple instructions.

```
mkdCodeChunkComplete(object=bioEr, message=c(quote(geneCounts <-
                  as.matrix(importData(system.file(
                  'extdata/BMDC_counts_FeatureCounts.xlsx',
                  package='easyreporting')))), quote(head(geneCounts, 20))),
                  sourceFilesList=system.file("script/importFunctions.R",
                  package="easyreporting"),
                  optionList=makeOptionsList(evalFlag=FALSE))
```

Note that the `mkdCodeChunkComplete` allows also to provide specific options for the CC which we are creating. In particular, in this case, we turned the `evalFlag=FALSE` because we already processed the step with the previous CC, so the code will not be evaluated.

3.3.2 CC2 rmarkdown side

The two above CC2 codes add the following CCs in the "rnaseq_report.rmd" file, which are identical. As remarked previously, the second CC has the `eval=FALSE` set as its argument. Moreover, this setting does not affects other global options of the report.

```
%# Loading Counts Data
%```{r eval=TRUE, echo=TRUE, warning=FALSE, message=FALSE, include=TRUE, cache=TRUE}
%source("importFunctions.R")
%geneCounts <- as.matrix(importData(system.file('extdata/BMDC_counts_FeatureCounts.xlsx',
%package='easyreporting')))
%head(geneCounts, 20)
%```
%
```

```
%```{r eval=FALSE, echo=TRUE, warning=FALSE, message=FALSE, include=TRUE, cache=TRUE}
%source("importFunctions.R")
%geneCounts <- as.matrix(importData(system.file('extdata/BMDC_counts_FeatureCounts.xlsx',
%package='easyreporting')))
%head(geneCounts, 20)
%```
```

3.3.3 CC2 report side

The above CC2 code corresponds to the part of the analysis report depicted in Figure S2.

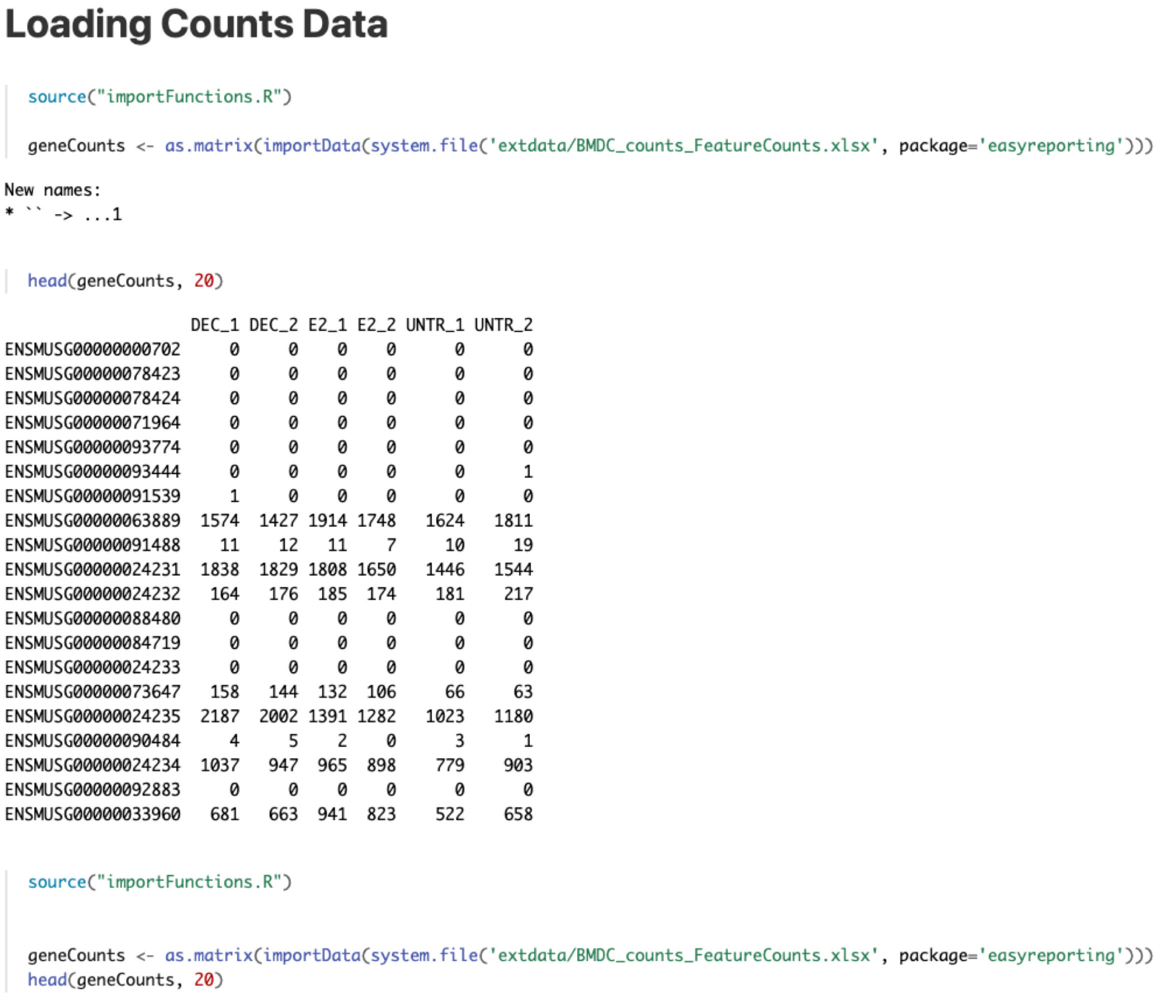

Loading Counts Data

```
source("importFunctions.R")

geneCounts <- as.matrix(importData(system.file('extdata/BMDC_counts_FeatureCounts.xlsx', package='easyreporting')))
```

```
New names:
* `` -> ...1
```

```
head(geneCounts, 20)
```

	DEC_1	DEC_2	E2_1	E2_2	UNTR_1	UNTR_2
ENSMUSG00000000702	0	0	0	0	0	0
ENSMUSG00000078423	0	0	0	0	0	0
ENSMUSG00000078424	0	0	0	0	0	0
ENSMUSG00000071964	0	0	0	0	0	0
ENSMUSG00000093774	0	0	0	0	0	0
ENSMUSG00000093444	0	0	0	0	0	1
ENSMUSG00000091539	1	0	0	0	0	0
ENSMUSG00000063889	1574	1427	1914	1748	1624	1811
ENSMUSG00000091488	11	12	11	7	10	19
ENSMUSG00000024231	1838	1829	1808	1650	1446	1544
ENSMUSG00000024232	164	176	185	174	181	217
ENSMUSG00000088480	0	0	0	0	0	0
ENSMUSG00000084719	0	0	0	0	0	0
ENSMUSG00000024233	0	0	0	0	0	0
ENSMUSG00000073647	158	144	132	106	66	63
ENSMUSG00000024235	2187	2002	1391	1282	1023	1180
ENSMUSG00000090484	4	5	2	0	3	1
ENSMUSG00000024234	1037	947	965	898	779	903
ENSMUSG00000092883	0	0	0	0	0	0
ENSMUSG00000033960	681	663	941	823	522	658

```
source("importFunctions.R")


geneCounts <- as.matrix(importData(system.file('extdata/BMDC_counts_FeatureCounts.xlsx', package='easyreporting')))
head(geneCounts, 20)
```

Figure S2: The report section produced by the CC2 R Markdown code

3.4 Data Counts Exploration

When analyzing omics data, it is a good practice to visualize a series of graphs to understand how the samples are distributed and if specific problems have to be faced during the analysis.

The box-plot of the log-counts is an example of the possible plots that can be used to compare the distribution of the samples. This plot can be easily obtained using the R *boxplot* function.

To trace this step in the report, the `mkdCodeChunkComplete` function can be used to insert the boxplot

function-call as the message to track in a new CC.

3.4.1 CC3 code side

The analyst can use the following code.

```
mkdTitle(bioEr, title="Plot Boxplot on count data", level=2)
mkdCodeChunkComplete(bioEr, message=paste0("boxplot(log(geneCounts+1),",
                        " col=c('red','red','orange','orange','purple','purple'),",
                        " main='Counts BoxPlot',las=1)"))
```

3.4.2 CC3 rmarkdown side

The above CC3 code adds the following CC in the “rnaseq_report.rmd” file.

```
% ## Plot Boxplot on count data
% ```{r eval=TRUE, echo=TRUE, warning=FALSE, message=FALSE, include=TRUE, cache=TRUE}
% boxplot(log(geneCounts+1),
% col=c('red','red','orange','orange','purple','purple'), main='Counts BoxPlot',las=1)
% ```
```

3.4.3 CC3 report side

The above CC3 code corresponds to the part of the analysis report depicted in Figure S3.

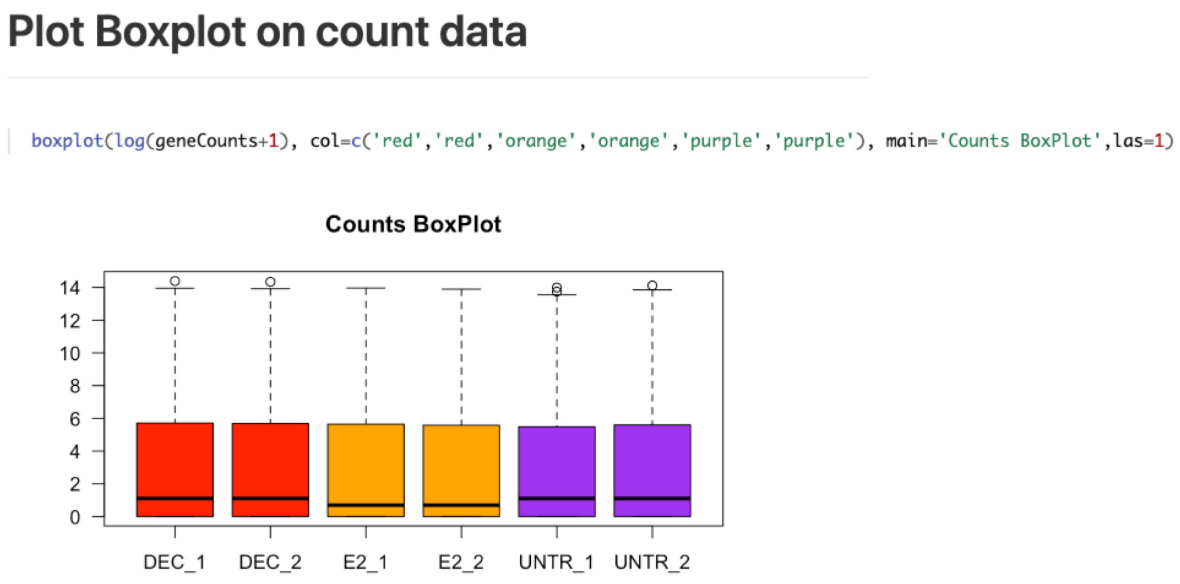

Figure S3: The report section produced by the CC3 R Markdown side code

In particular, in this case, the figure shows that each sample has a great number of genes with 0 counts, suggesting that suitable filtering procedures have to be applied to the data before further processing.

3.5 Filtering and Normalization steps

To perform the filtering step, we use the `filtered.data` function present in the *NOISeq* package. After that, we can normalize the data across the samples using the `betweenLaneNormalization` from the *EDASeq* package and display the Principal Component Analysis (PCA) by using the `plotPCA` function of the *DESeq2* package.

To trace all function calls we need to repeatedly use the `mkdCodeChunkComplete` function for consecutive CCs.

3.5.1 CC4 code side

The analyst can use the following code.

```
mkdTitle(bioEr, title="Filtering Low Abundant Features", level=1)
mkdCodeChunkComplete(object=bioEr, message=paste("fgeneCounts <- ",
              "NOISeq::filtered.data(dataset=geneCounts, ",
              "factor=c('D', 'D', 'E', 'E', 'C', 'C'), norm=FALSE, method=3, cv.cutoff=100, cpm=0.5)",
              "boxplot(log(fgeneCounts+1),col=c('red','red','orange','orange','purple','purple'), ",
              "main='Counts BoxPlot',las=1)", sep="\n"))
```

3.5.2 CC4 rmarkdown side

The above CC4 code adds the following CC in the "rnaseq_report.rmd" file.

```
% # Filtering Low Abundant Features
% ```{r eval=TRUE, echo=TRUE, warning=FALSE, message=FALSE, include=TRUE, cache=TRUE}
% fgeneCounts <-
% NOISeq::filtered.data(dataset=geneCounts,
% factor=c('D', 'D', 'E', 'E', 'C', 'C'), norm=FALSE, method=3, cv.cutoff=100, cpm=0.5)
% boxplot(log(fgeneCounts+1),col=c('red','red','orange','orange','purple','purple'),
% main='Counts BoxPlot',las=1)
% ```
```

3.5.3 CC4 report side

The above CC4 code corresponds to the part of the analysis report depicted in Figure S4.

3.5.4 CC5 code side

For the normalization and the PCA, we implemented the following code.

```
mkdTitle(bioEr, title="Normalizing Features Across Samples", level=1)
mkdCodeChunkComplete(object=bioEr, message=paste("nfgeneCounts <- ",
              "EDASeq::betweenLaneNormalization(fgeneCounts, which='upper')", sep="\n")

mkdTitle(bioEr, title="Plot PCA on count data", level=2)
mkdCodeChunkComplete(bioEr, message=paste("se <- SummarizedExperiment((log2(nfgeneCounts)+ 1), ",
                "colData=DataFrame(rownames=colnames(nfgeneCounts), " ,
                "condition=c('DEC', 'DEC', 'E2', 'E2', 'CTRL', 'CTRL')))",
                "DESeq2::plotPCA(DESeqTransform(se))", sep="\n"))
```

3.5.5 CC5 rmarkdown side

The above CC5 code adds the following CC in the "rnaseq_report.rmd" file.

```
% # Normalizing Features Across Samples
% ```{r eval=TRUE, echo=TRUE, warning=FALSE, message=FALSE, include=TRUE, cache=TRUE}
% nfgeneCounts <- EDASeq::betweenLaneNormalization(fgeneCounts, which='upper')
% ```
% ## Plot PCA on count data
% ```{r eval=TRUE, echo=TRUE, warning=FALSE, message=FALSE, include=TRUE, cache=TRUE}
%
% se <- SummarizedExperiment((log2(nfgeneCounts)+ 1),
```

Filtering Low Abundant Features

```
fgeneCounts <- NOISeq::filtered.data(dataset = geneCounts, factor = c("D",
    "D", "E", "E", "C", "C"), norm = FALSE, method = 3, cv.cutoff = 100,
    cpm = 0.5)
```

```
Filtering out low count features...
13390 features are to be kept for differential expression analysis with filtering method 3
```

```
boxplot(log(fgeneCounts + 1), col = c("red", "red", "orange",
    "orange", "purple", "purple"), main = "Counts BoxPlot", las = 1)
```

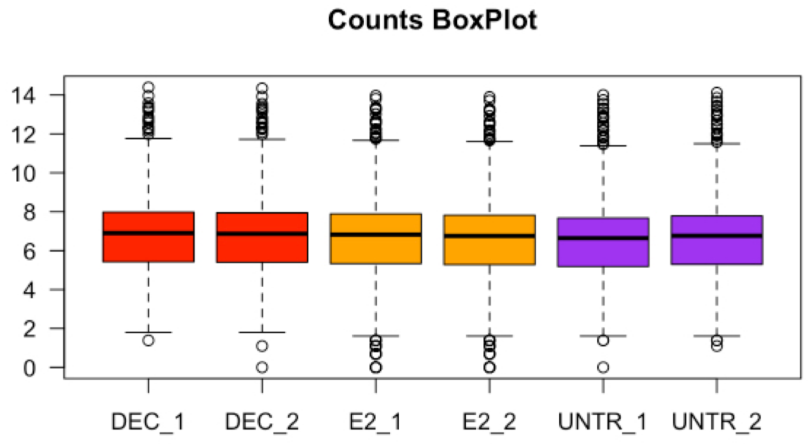

Figure S4: The report section produced by the CC4 R Markdown side code

```
% colData=DataFrame(rownames=colnames(nfgeneCounts),
% condition=c('DEC', 'DEC', 'E2', 'E2', 'CTRL', 'CTRL')))
%
% DESeq2::plotPCA(DESeqTransform(se))
% ```
```

3.5.6 CC5 report side

The above CC5 code corresponds to the part of the analysis report depicted in Figure S5.

Normalizing Features Across Samples

```
nfgeneCounts <- EDASeq::betweenLaneNormalization(fgeneCounts, which='upper')
```

Plot PCA on count data

```
se <- SummarizedExperiment::SummarizedExperiment((log2(nfgeneCounts)+ 1),
colData=S4Vectors::DataFrame(rownames=colnames(nfgeneCounts),
condition=c('DEC', 'DEC', 'E2', 'E2', 'CTRL', 'CTRL')))
DESeq2::plotPCA(DESeq2::DESeqTransform(se))
```

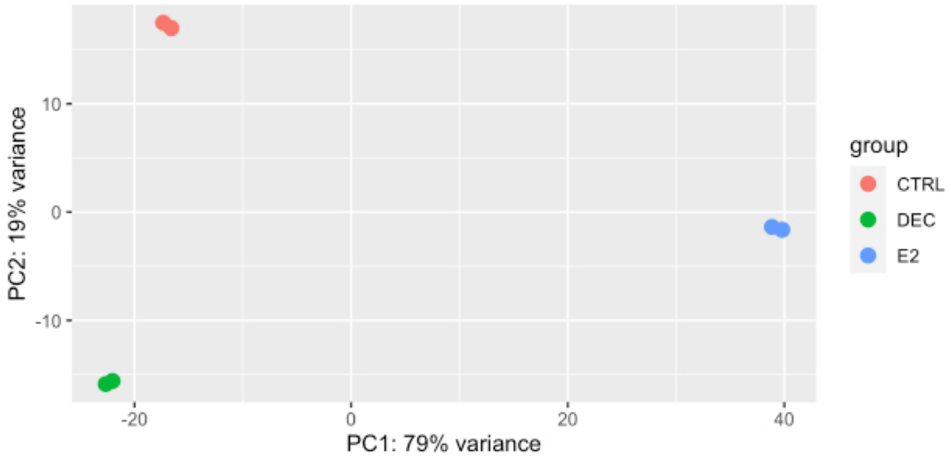

Figure S5: The report section produced by the CC5 R Markdown side code

In particular, in this case, the figures show that the replicates are correctly associated and the treatments are correctly grouped. It is possible to insert comments related to specific outputs as shown in the following CC.

3.6 Differential Expression Analysis

Assuming that the data were correctly normalized, it is possible to perform the differential expression gene (DEG) analysis by using the *edgeR* package. To simplify such step, we assume to have a user-defined function named `applyEdgeREx()` stored in the *geneFunctions.R* that implements the required test.

Moreover, instead of using the `mkdCodeChunkComplete()`, here the `mkdCodeChunkCommented()` function can be used to allow to add a comment preceeding the code chunk using the `commentMsg` parameter.

For this analysis, we first tested the differences between DEC and Control, then E2 and Control conditions.

3.6.1 CC6 code side

This can be implemented with the following code.

```
mkdCodeChunkTitledCommented(bioEr, title="Differential Expression Analysis",
                codeMsg=paste0("degList <- applyEdgeREx(counts=nfgeneCounts, ",
                "factors=c('DEC', 'DEC', 'E2', 'E2', 'UNTR', 'UNTR'), ",
                "contrasts=c('DEC - UNTR', 'E2 - UNTR'),",
                "p.threshold=1)"),
            commentMsg=paste("As we saw from the PCA, the groups are well separated, ",
            "so we can perform a Differential Expression analysis with edgeR.", sep="\n"),
            sourceFilesList=system.file("script/geneFunctions.R", package="easyreporting"))
```

3.6.2 CC6 rmarkdown side

The above CC6 code adds the following CC in the “rnaseq_report.rmd” file.

```
% # Differential Expression Analysis
% As we saw from the PCA, the groups are well separated,
% so we can perform a Differential Expression analysis with edgeR.
% ```{r eval=TRUE, echo=TRUE, warning=FALSE, message=FALSE, include=TRUE, cache=TRUE}
% source("geneFunctions.R")
% degList <- applyEdgeREx(counts=nfgeneCounts,
% factors=c('DEC', 'DEC', 'E2', 'E2', 'UNTR', 'UNTR'),
% contrasts=c('DEC - UNTR', 'E2 - UNTR'), p.threshold=1)
% ```
```

3.6.3 CC6 report side

The above CC6 code corresponds to the part of the analysis report depicted in Figure S6.

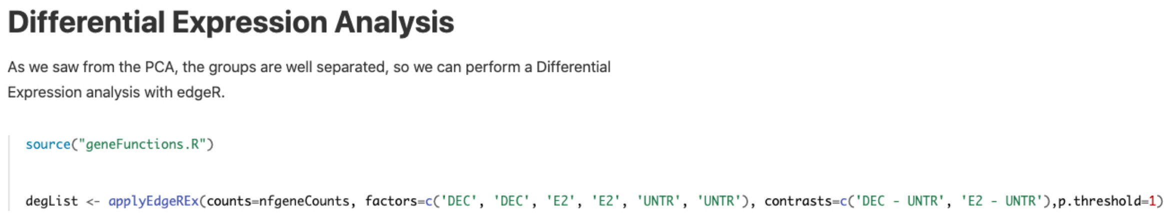

Figure S6: The report section produced by the CC6 R Markdown side code

3.7 Differential Expressed Genes Inspection

The output of a DEG Analysis can be graphically represented by an MA-plot (or a Volcano plot) for each investigated contrast.

In this example, we can use the MA-plot and produce the graphic by using the `plot` function where to depict on the x-axis the log of the Counts per Million (CPM) and on the y-axis the log of the Fold Change (FC) computed by *edgeR* during the DEG analysis. In the following plot, the genes are represented by the dots, in black the not significant genes and in red the significant DEGs.

With *easyreporting*, by the aid of `mkdCodeChunkComplete`, it is possible to create a new title and a new chunk within one singular function-call.

3.7.1 CC7 code side

The user can use the following code.

```
mkdCodeChunkTitledCommented(bioEr, title="MA-Plot", level=2,
                            code="MAedgeRMAPlotEx(degList=degList)",
                            sourceFilesList=system.file("script/plotFunctions.R",
                                                        package="easyreporting"))
```

3.7.2 CC7 rmarkdown side

The above CC7 code adds the following CC in the “rnaseq_report.rmd” file.

```
% ## MA Plot of DEGs
% ```{r eval=TRUE, echo=TRUE, warning=FALSE, message=FALSE, include=TRUE, cache=TRUE}
%
% source("plotFunctions.R")
% MAedgeRMAPlotEx(degList=degList)
% ```
```

3.7.3 CC7 report side

The above CC7 code corresponds to the part of the analysis report depicted in Figure S7.

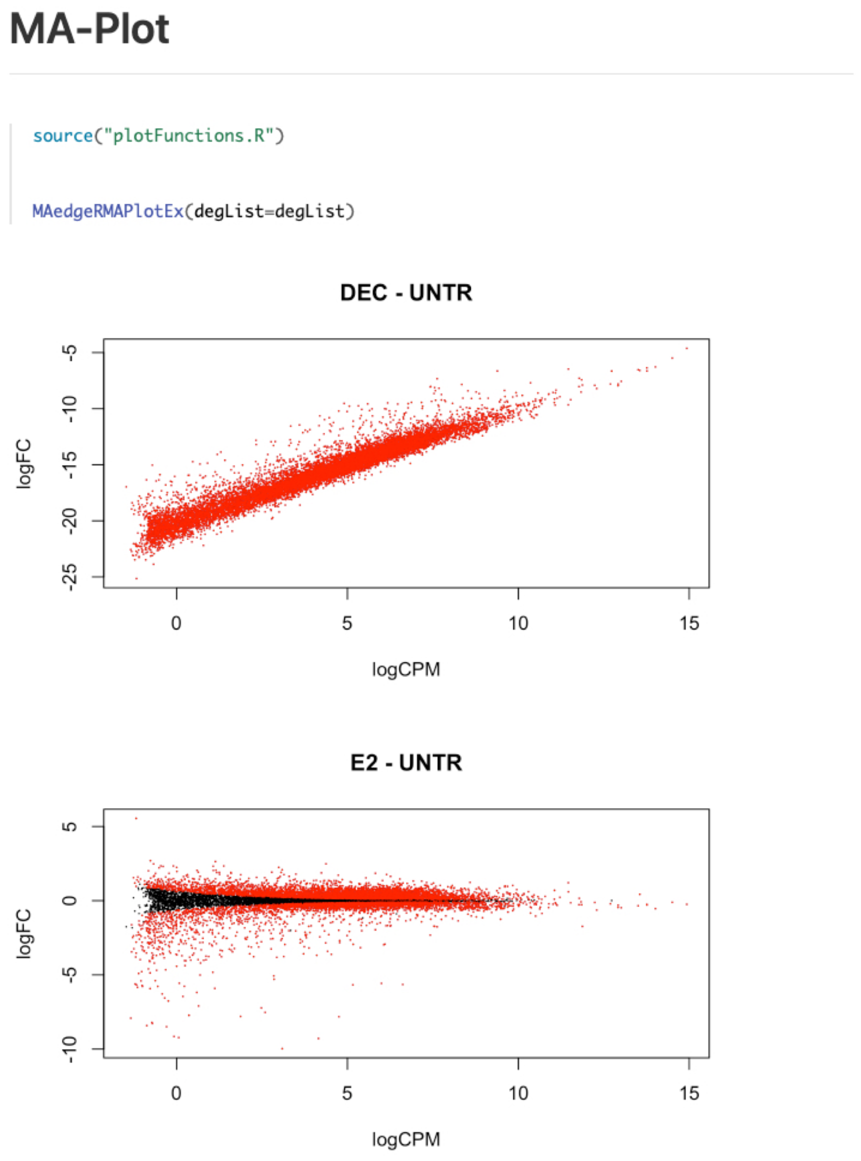

Figure S7: The report section produced by the CC7 R Markdown side code

3.8 DEGs Comparison

To compare the DEGs between the two main conditions (DEC and E2) we can use a Venn Diagram, where each circle corresponds to the differentially expressed genes of a specific comparison.

3.8.1 CC8 code side

The user can use the following code.

```
mkdTitle(bioEr, "DEGs Venn Diagram", level=2)
mkdCodeChunkComplete(bioEr, message=paste("limma::vennDiagram(",
                    "limma::vennCounts(cbind(degList[[1]]$FDR < 0.01, ",
```

```
            "degList[[2]]$FDR < 0.01)), names=c('DEC', 'E2'))",sep="\n"))
```

3.8.2 CC8 rmarkdown side

The above CC8 code adds the following CC in the “rnaseq_report.rmd” file.

```
% ### DEGs Venn Diagram
% ```{r eval=TRUE, echo=TRUE, warning=FALSE, message=FALSE, include=TRUE, cache=TRUE}
% limma::vennDiagram(limma::vennCounts(
% cbind(degList[[1]]$FDR < 0.01, degList[[2]]$FDR < 0.01)), names=c('DEC', 'E2'))
% ```
```

3.8.3 CC8 report side

The above CC8 code corresponds to the part of the analysis report depicted in Figure S8.

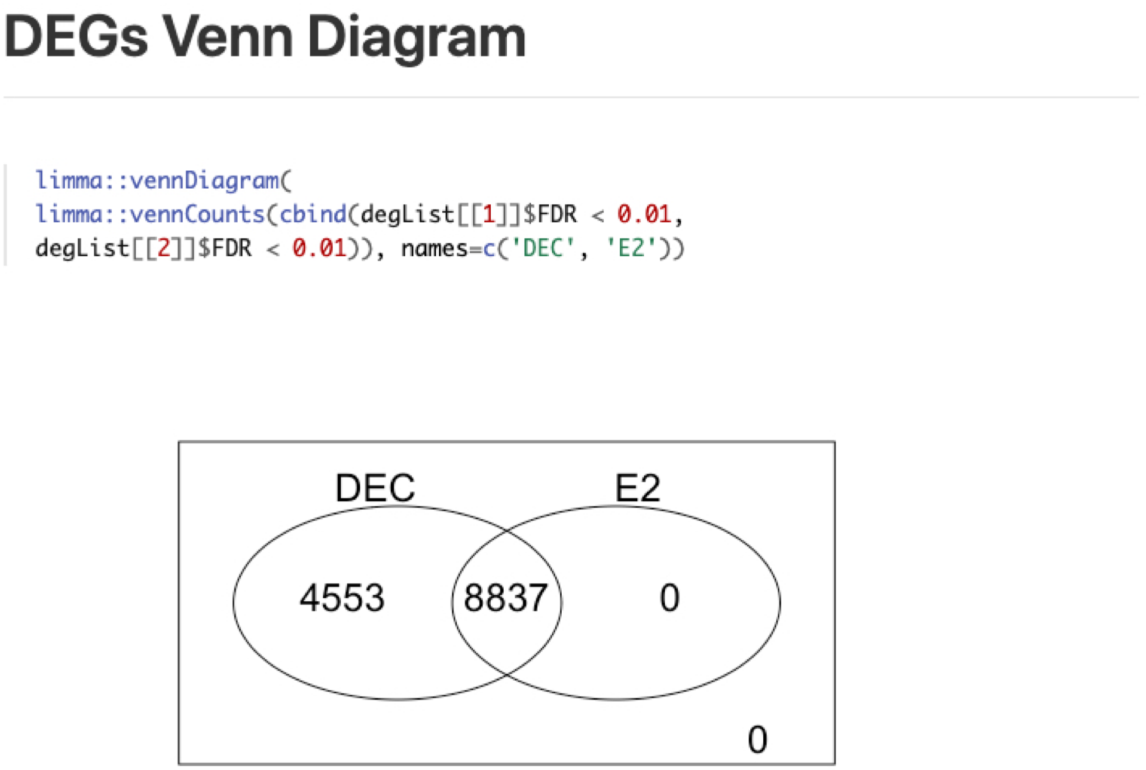

Figure S8: The report section produced by the CC8 R Markdown side code

3.9 Report Compilation

Once the analysis is completed, the package allows to compile the produced “rnaseq_report.rmd” report simply by using the `compile()` method. The `compile` method produces the final report (in the user defined format) and automatically appends to the report end a CC with the external available resources previously set with the `addResource` method and another CC with the sessionInfo() to trace all the package versions used for the analysis.

3.9.1 CC9 code side

To compile the report, the analyst can use the following code.

```
bioEr <- addResource(bioEr, source="GEO", "GSE60231", "Transcriptome of BMDC to different antigen delive[illegible]
compile(bioEr)
```

3.9.2 CC9 rmarkdown side

The above CC9 code adds the following CC in the “rnaseq_report.rmd” file.

```
% # Session Info
%
% ```{r eval=TRUE, echo=TRUE, warning=FALSE, message=FALSE, include=TRUE, cache=TRUE}
% sessionInfo()
% ```
```

3.9.3 CC9 report side

The above CC9 code compiles the entire report and automatically adds at the end of the report the Resource Availability and the Session Info sections as in the example in Figure S9.

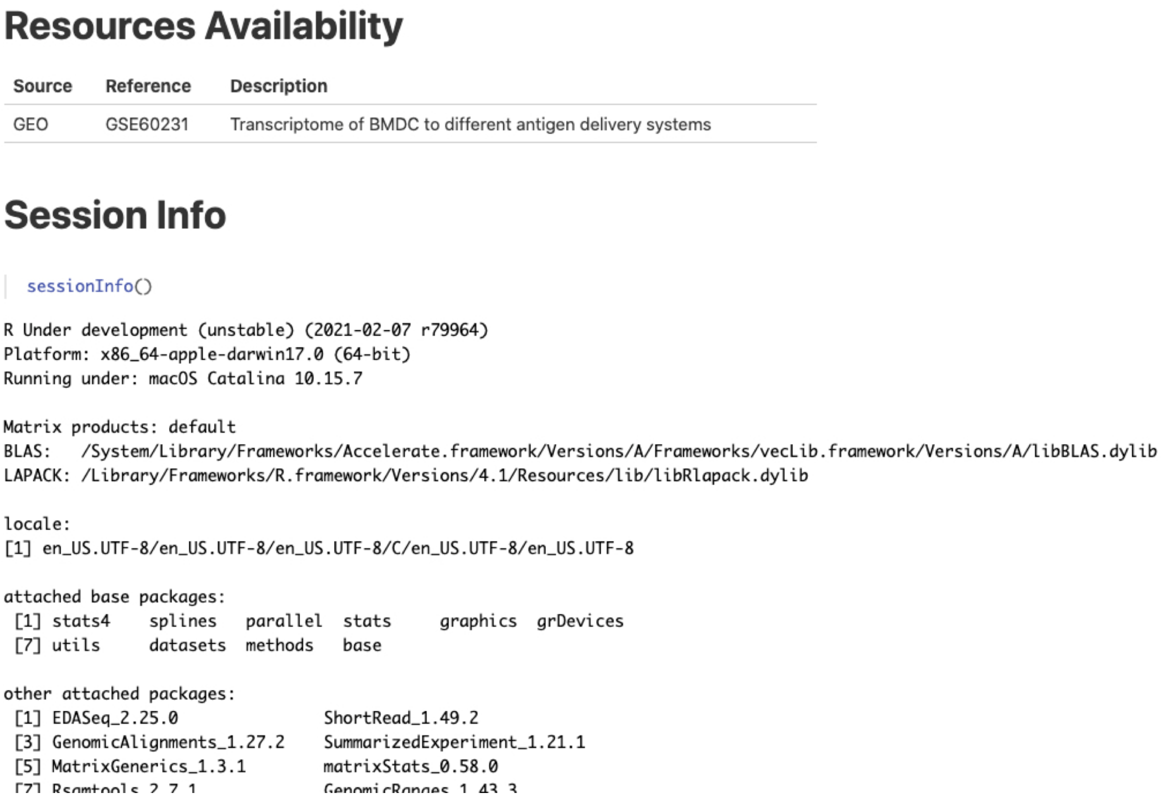

Resources Availability

Source	Reference	Description
GEO	GSE60231	Transcriptome of BMDC to different antigen delivery systems

Session Info

```
sessionInfo()
```

```
R Under development (unstable) (2021-02-07 r79964)
Platform: x86_64-apple-darwin17.0 (64-bit)
Running under: macOS Catalina 10.15.7

Matrix products: default
BLAS:   /System/Library/Frameworks/Accelerate.framework/Versions/A/Frameworks/vecLib.framework/Versions/A/libBLAS.dylib
LAPACK: /Library/Frameworks/R.framework/Versions/4.1/Resources/lib/libRlapack.dylib

locale:
[1] en_US.UTF-8/en_US.UTF-8/en_US.UTF-8/C/en_US.UTF-8/en_US.UTF-8

attached base packages:
 [1] stats4    splines   parallel  stats     graphics  grDevices
 [7] utils     datasets  methods   base

other attached packages:
 [1] EDASeq_2.25.0               ShortRead_1.49.2
 [3] GenomicAlignments_1.27.2    SummarizedExperiment_1.21.1
 [5] MatrixGenerics_1.3.1        matrixStats_0.58.0
 [7] Rsamtools_2.7.1             GenomicRanges_1.43.3
```

Figure S9: The report section produced by the CC9 R Markdown side code

4 Running easyreporting example GUI

To provide an example of GUI that incorporates the *easyreporting* package, we defined one of them into the package since its version 1.3.1.

To run it just run the following code:

```
erGUIVolcano()
```

References

Costa, Valerio, Dario Righelli, Francesco Russo, Piergiuseppe De Berardinis, Claudia Angelini, and Luciana

D'Apice. 2017. "Distinct Antigen Delivery Systems Induce Dendritic Cells' Divergent Transcriptional Response: New Insights from a Comparative and Reproducible Computational Analysis." *International Journal of Molecular Sciences* 18 (3): 494.
