# Supplementary material for: Easyreporting simplifies the implementation of Reproducible Research layers in R software
Source: PLoS One. 2021 May 10;16(5):e0244122. doi: 10.1371/journal.pone.0244122 (PMC8109797; doi:10.1371/journal.pone.0244122)
Supplement: S2 File — (PDF) [file pone.0244122.s002.pdf]

# RNA-seq Analysis Report

AUTHORS

Dario Righelli fake_email@gmail.com

Claudia Angelini

AFFILIATIONS

Institute of Applied Mathematics, CNR, Naples, IT

Institute of Applied Mathematics, CNR, Naples, IT

PUBLISHED

March 15, 2021

## Loading Counts Data

```
source ("importFunctions.R")

geneCounts <- as.matrix(importData(system.file(
'extdata/BMDC_counts_FeatureCounts.xlsx', package='easyreporting'))
)
```

```
Loading required package: readxl

New names:
* `` -> ...1
```

```
head (geneCounts, 20 )
```

```
                   DEC_1 DEC_2 E2_1 E2_2 UNTR_1 UNTR_2
ENSMUSG00000000702     0     0    0    0      0      0
ENSMUSG00000078423     0     0    0    0      0      0
ENSMUSG00000078424     0     0    0    0      0      0
ENSMUSG00000071964     0     0    0    0      0      0
ENSMUSG00000093774     0     0    0    0      0      0
ENSMUSG00000093444     0     0    0    0      0      1
ENSMUSG00000091539     1     0    0    0      0      0
ENSMUSG00000063889  1574  1427 1914 1748   1624   1811
ENSMUSG00000091488    11    12   11    7     10     19
ENSMUSG00000024231  1838  1829 1808 1650   1446   1544
ENSMUSG00000024232   164   176  185  174    181    217
ENSMUSG00000088480     0     0    0    0      0      0
ENSMUSG00000084719     0     0    0    0      0      0
ENSMUSG00000024233     0     0    0    0      0      0
ENSMUSG00000073647   158   144  132  106     66     63
ENSMUSG00000024235  2187  2002 1391 1282   1023   1180
ENSMUSG00000090484     4     5    2    0      3      1
ENSMUSG00000024234  1037   947  965  898    779    903
```

```
ENSMUSG00000092883     0     0    0    0      0      0
ENSMUSG00000033960   681   663  941  823    522    658
```

```
source  ( "importFunctions.R")

geneCounts <- as.matrix( importData( system.file(
"extdata/BMDC_counts_FeatureCounts.xlsx",
    package = "easyreporting") ) )
head ( geneCounts, 20 )
```

## Plot Boxplot on count data

```
boxplot ( log ( geneCounts+ 1 ) , col= c
( 'red' ,'red' ,'orange' ,'orange' ,'purple' ,'purple' ) , main=
'Counts BoxPlot' ,las= 1 )
```

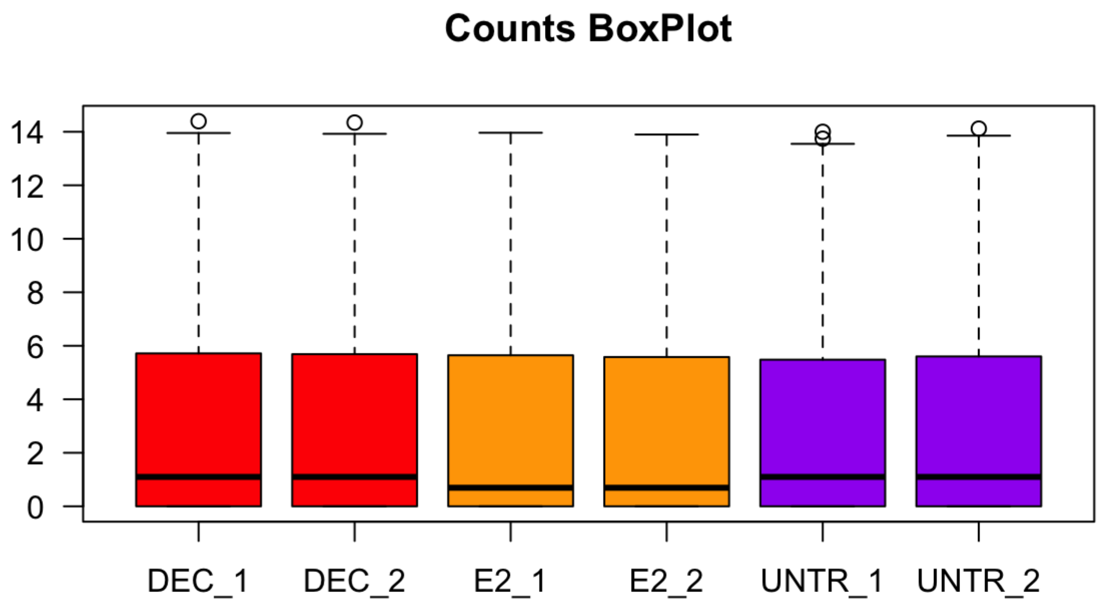


# Filtering Low Abundant Features

```
fgeneCounts <- NOISeq :: filtered.data( dataset = geneCounts,
factor = c ( "D" ,
    "D" , "E" , "E" , "C" , "C" ) , norm = FALSE ,
method = 3 , cv.cutoff = 100 ,
    cpm = 0.5 )
```

```
Filtering out low count features...
```

```
13390 features are to be kept for differential expression analysis with filtering method 3
```

```
boxplot ( log ( fgeneCounts + 1 ) , col = c ( "red" , "red" , "orange" ,
"orange" , "purple" , "purple" ) , main = "Counts BoxPlot" , las =
1 )
```

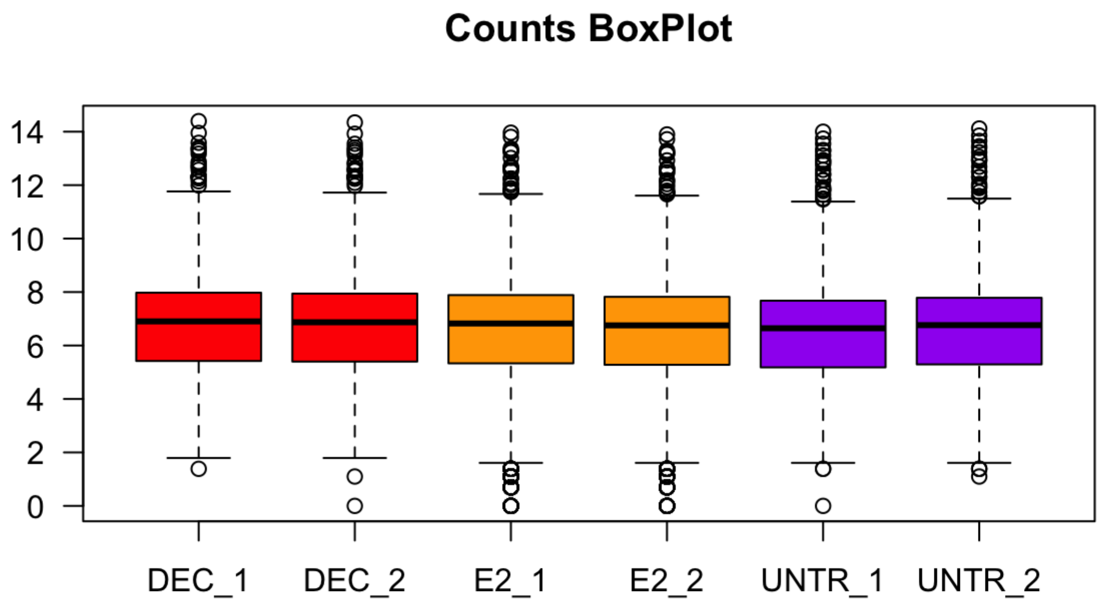


# Normalizing Features Across Samples

```
nfgeneCounts <- EDASeq :: betweenLaneNormalization( fgeneCounts, which
= 'upper' )
```

## Plot PCA on count data

```
se <- SummarizedExperiment:: SummarizedExperiment( (
log2 ( nfgeneCounts) + 1 ) ,
colData= S4Vectors:: DataFrame( rownames= colnames (
nfgeneCounts) ,
condition= c ( 'DEC' , 'DEC' , 'E2' , 'E2' , 'CTRL' ,
'CTRL' ) ) )
DESeq2 :: plotPCA ( DESeq2 :: DESeqTransform( se )
)
```

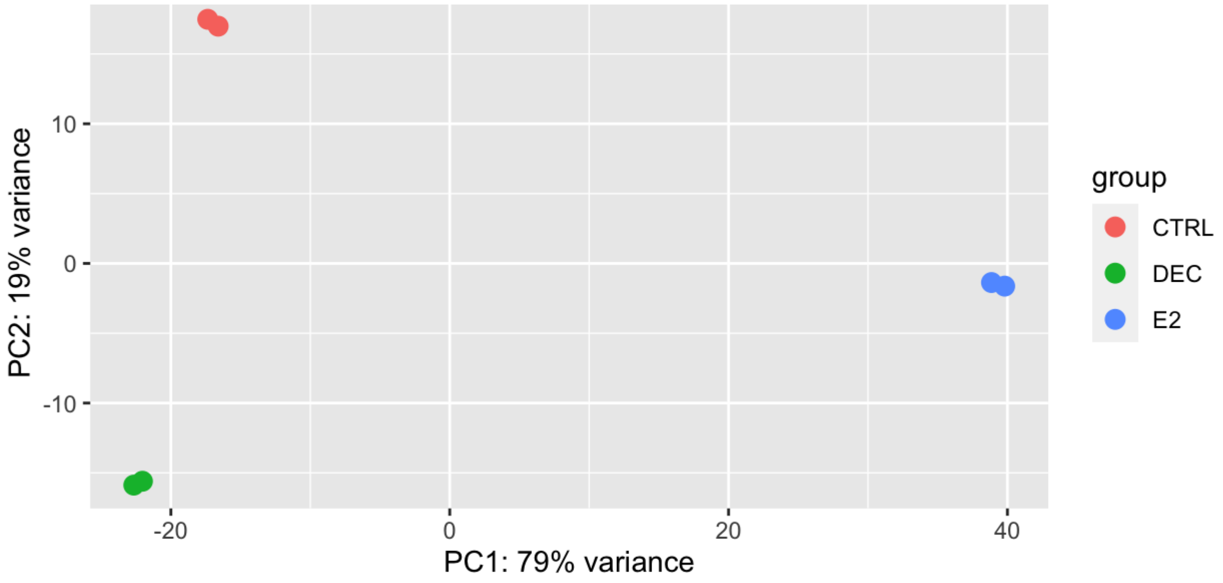


# Differential Expression Analysis

As we saw from the PCA, the groups are well separated, so we can perform a Differential Expression analysis with edgeR.

```
source    (        "geneFunctions.R")


degList   <-        applyEdgeREx(        counts=        nfgeneCounts, factors=
c          (        'DEC'    , 'DEC'    , 'E2'     , 'E2'     , 'UNTR'   , 'UNTR'   )          ,
contrasts=          c         (         'DEC - UNTR'  , 'E2 - UNTR'   )          ,p.threshold
=          1          )
```

## MA-Plot

```
source    (        "plotFunctions.R")


MAedgeRMAPlotEx(        degList=        degList  )
```

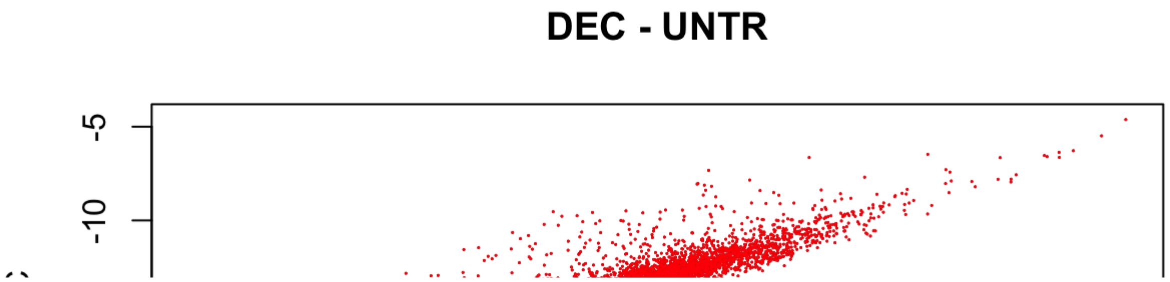

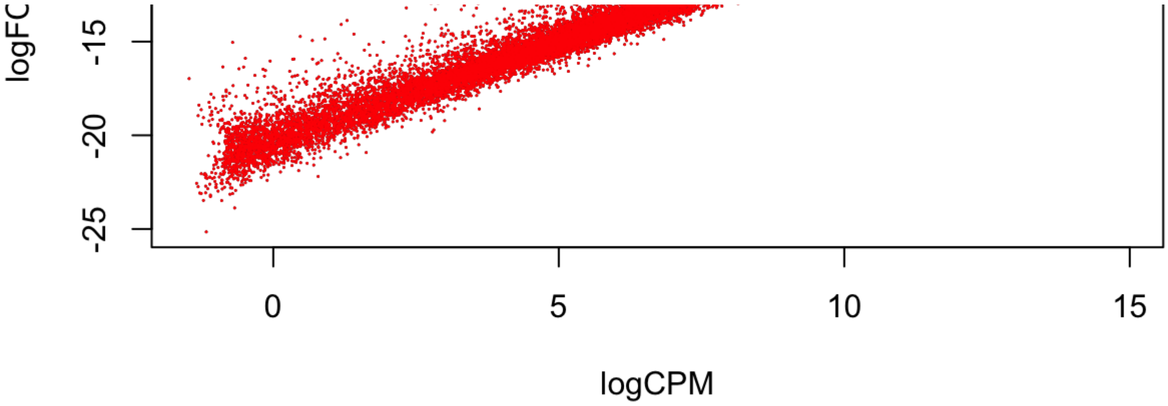


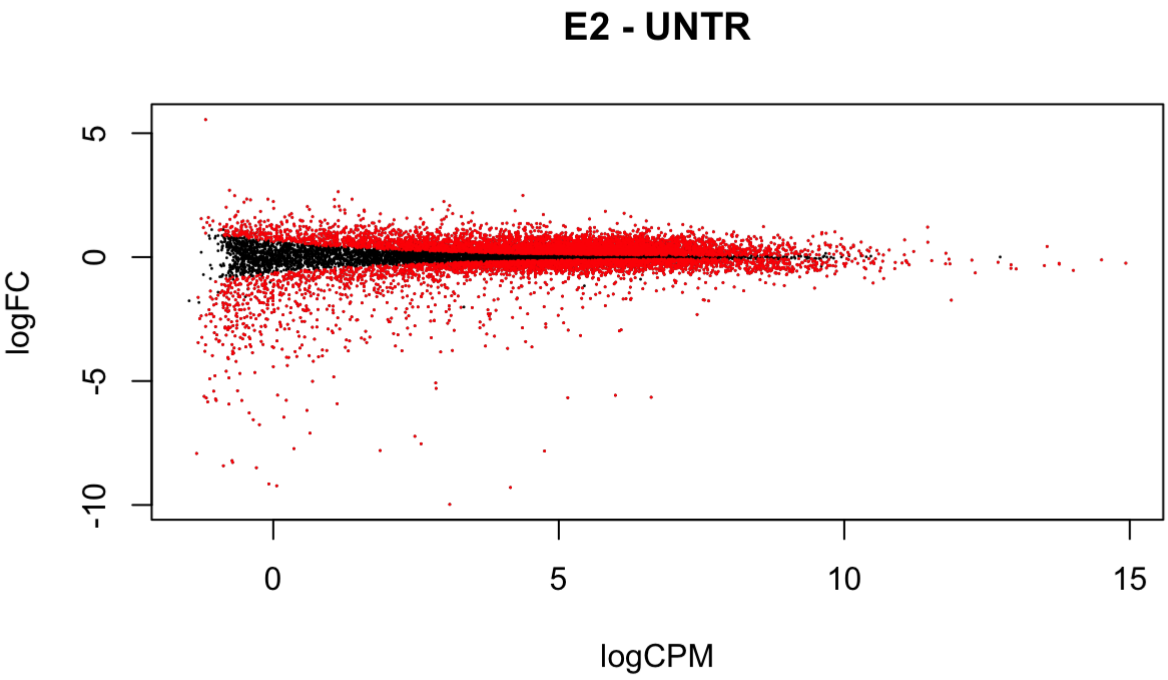


## DEGs Venn Diagram

## DEGs Venn Diagram

```
limma :: vennDiagram(
limma :: vennCounts( cbind ( degList [[ 1 ]
] $ FDR < 0.01 ,
degList [[ 2 ] ] $ FDR < 0.01 )
) , names= c ( 'DEC' , 'E2' ) )
```

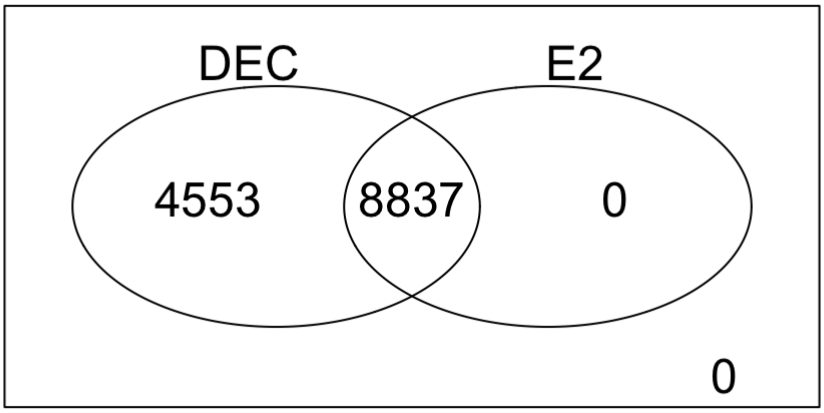


# Resources Availability

| Source | Reference | Description |
|---|---|---|
| GEO | GSE60231 | Transcriptome of BMDC to different antigen delivery systems |

# Session Info

```
sessionInfo( )
```

```
R Under development (unstable) (2021-02-07 r79964)
Platform: x86_64-apple-darwin17.0 (64-bit)
Running under: macOS Catalina 10.15.7

Matrix products: default
BLAS:   /System/Library/Frameworks/Accelerate.framework/Versions/A/Frameworks/vecLib.framewo
LAPACK: /Library/Frameworks/R.framework/Versions/4.1/Resources/lib/libRlapack.dylib

locale:
```

```
[1] en_US.UTF-8/en_US.UTF-8/en_US.UTF-8/C/en_US.UTF-8/en_US.UTF-8

attached base packages:
[1] stats     graphics  grDevices utils     datasets  methods
[7] base

other attached packages:
[1] readxl_1.3.1        easyreporting_1.3.2 distill_1.2

loaded via a namespace (and not attached):
  [1] colorspace_2.0-0            rjson_0.2.20
  [3] hwriter_1.3.2               ellipsis_0.3.1
  [5] XVector_0.31.1              GenomicRanges_1.43.3
  [7] rstudioapi_0.13             farver_2.1.0
  [9] bit64_4.0.5                 AnnotationDbi_1.53.1
 [11] fansi_0.4.2                 xml2_1.3.2
 [13] codetools_0.2-18            splines_4.1.0
 [15] R.methodsS3_1.8.1           downlit_0.2.1
 [17] cachem_1.0.4                geneplotter_1.69.0
 [19] knitr_1.31                  jsonlite_1.7.2
 [21] Rsamtools_2.7.1             annotate_1.69.0
 [23] dbplyr_2.1.0                png_0.1-7
 [25] R.oo_1.24.0                 compiler_4.1.0
 [27] httr_1.4.2                  assertthat_0.2.1
 [29] Matrix_1.3-2                fastmap_1.1.0
 [31] limma_3.47.8                htmltools_0.5.1.1
 [33] prettyunits_1.1.1           tools_4.1.0
 [35] gtable_0.3.0                glue_1.4.2
 [37] GenomeInfoDbData_1.2.4      dplyr_1.0.5

 [39] rappdirs_0.3.3              ShortRead_1.49.2
 [41] Rcpp_1.0.6                  Biobase_2.51.0
 [43] cellranger_1.1.0            jquerylib_0.1.3
 [45] vctrs_0.3.6                 Biostrings_2.59.2
 [47] rtracklayer_1.51.5          xfun_0.22
 [49] stringr_1.4.0               lifecycle_1.0.0
 [51] restfulr_0.0.13             statmod_1.4.35
 [53] XML_3.99-0.5                edgeR_3.33.3
 [55] zlibbioc_1.37.0             scales_1.1.1
 [57] aroma.light_3.21.0          hms_1.0.0
 [59] MatrixGenerics_1.3.1        parallel_4.1.0
 [61] SummarizedExperiment_1.21.1 RColorBrewer_1.1-2
 [63] yaml_2.2.1                  curl_4.3
 [65] memoise_2.0.0               ggplot2_3.3.3
 [67] sass_0.3.1                  biomaRt_2.47.5
 [69] latticeExtra_0.6-29         stringi_1.5.3
 [71] RSQLite_2.2.3               highr_0.8
 [73] genefilter_1.73.1           S4Vectors_0.29.7
 [75] BiocIO_1.1.2                GenomicFeatures_1.43.4
 [77] BiocGenerics_0.37.1         filelock_1.0.2
 [79] BiocParallel_1.25.4         GenomeInfoDb_1.27.6
 [81] rlang_0.4.10                pkgconfig_2.0.3
 [83] matrixStats_0.58.0          bitops_1.0-6
 [85] evaluate_0.14               lattice_0.20-41
```

```
 [87] purrr_0.3.4                  labeling_0.4.2
 [89] GenomicAlignments_1.27.2     bit_4.0.4
 [91] tidyselect_1.1.0             magrittr_2.0.1
 [93] DESeq2_1.31.14               R6_2.5.0
 [95] IRanges_2.25.6               generics_0.1.0
 [97] DelayedArray_0.17.9          DBI_1.1.1
 [99] pillar_1.5.1                 survival_3.2-7
[101] KEGGREST_1.31.1              RCurl_1.98-1.2
[103] tibble_3.1.0                 EDASeq_2.25.0
[105] crayon_1.4.1                 utf8_1.1.4
[107] BiocFileCache_1.15.1         rmarkdown_2.7
[109] jpeg_0.1-8.1                 progress_1.2.2
[111] locfit_1.5-9.4               grid_4.1.0
[113] blob_1.2.1                   digest_0.6.27
[115] xtable_1.8-4                 R.utils_2.10.1
[117] openssl_1.4.3                stats4_4.1.0
[119] munsell_0.5.0                NOISeq_2.35.0
[121] bslib_0.2.4                  askpass_1.1
```
